# In Vivo Evaluation of Conductive Biopolymer‐Based 3D Bioprinted Nerve Conduit in Sciatic Nerve Injury Repair

**DOI:** 10.1002/mabi.70223

**Published:** 2026-07-18

**Authors:** Nasera Rizwana, Yogesh H S, Kaustubh Raundal, Janani Sriramakrishnan, Shounak De, Syed Sahal, Goutam Thakur, Ashwath Acharya, Vipul Agarwal, Manasa Nune

**Affiliations:** ^1^ Manipal Institute of Regenerative Medicine Manipal Academy of Higher Education Manipal Karnataka India; ^2^ Department of Pharmacology Bangalore Campus NITTE College of Pharmaceutical Sciences (NITTE Deemed to Be University) Bangalore Karnataka India; ^3^ Manipal Institute of Technology Manipal Academy of Higher Education Manipal Karnataka India; ^4^ Department of Hand Surgery Kasturba Medical College Manipal Academy of Higher Education Manipal Karnataka India; ^5^ Department of Materials Science and Engineering Monash University Clayton Victoria Australia

**Keywords:** 3D bioprinting, alginate, methylcellulose, peripheral nerve injury, reduced graphene oxide

## Abstract

Peripheral nerve injury (PNI) is one of the most common conditions that occurs due to trauma and accidents. A successful peripheral nerve regeneration can potentially benefit from the use of conduits with adequate physical, mechanical, and biochemical cues to recapitulate the native neural microenvironment. We report an electroconductive 3D bioprinted nerve conduit fabricated using alginate/methylcellulose/reduced graphene oxide (Alg/MC/rGO) hydrogel. The incorporation of rGO rendered the hydrogel with improved electrical conductivity while maintaining good printability and shape fidelity through dual crosslinking using calcium chloride. rGO was characterized thoroughly to confirm its physicochemical characteristics. The optimized Alg/MC/rGO hydrogel showed shear‐thinning behaviour required for extrusion bioprinting and 3D structures (grid and nerve conduit) were successfully printed. Physicochemical analysis of Alg/MC/rGO scaffolds confirmed the presence of rGO within the scaffolds. On analysing conductivity properties, it was observed that Alg/MC/rGO scaffolds showed conductivity values (∼7.5 × 10^−2^ S/m) within the physiological range of the peripheral nerve. In vitro assays such as MTT and Live/dead assay confirmed that the scaffolds showed cytocompatibility above the threshold as mentioned in ISO standards. In vivo evaluation using sciatic nerve transection (5 mm) model in Sprague Dawley rats showed that Alg/MC/rGO nerve conduits significantly enhanced sciatic functional index values at 21‐day post‐implantation. Histological analyses further confirmed good nerve fiber alignment, remyelination and reduced collagen percentage in the regenerated nerve. Overall, these findings establish that incorporation of rGO within Alg/MC hydrogel along with a dual crosslinking strategy provided structural and conductive cues to the nerve conduit that accelerated nerve repair thereby providing a promising platform for next‐generation nerve guide conduit (NGC).

## Graphical abstract

This study presents the first report of fabricating and characterizing an extrusion‐based 3D bioprinted nerve conduit using a blend of reduced graphene oxide within an alginate/methylcellulose hydrogel system.

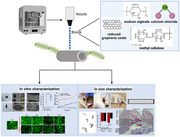

## 1 Introduction

Peripheral nerve injury affects a million people globally every year resulting in sensory and motor impairment of nerves leading to loss of function and disability thereby greatly affecting the patient's life. Although nerve anastomosis has been considered as one of the treatment strategies for smaller defects, larger defects have significantly fewer therapeutic options. Currently, the gold standard treatment for treating PNI is autografts. The use of autografts is limited due to its disadvantages including donor scarcity, differences in size and geometry of donor nerves, secondary deformities in donor areas, and risk of developing neuromas [1, 2]. Although, peripheral nervous system can regenerate, functional recovery of the injured nerves in cases of neurotmesis does not reach the preinjury level. The presence of an appropriate microenvironment that will help the regenerating axons to form bands of Bunger is of utmost importance to restore the function of the injured nerve [3, 4]. Owing to the limitations of autografts, nerve guidance conduits are being considered as a potential alternative [5, 6].

Tissue‐engineered tubular constructs made of biopolymers, designed to provide necessary physical, mechanical, or biochemical cues for nerve regeneration are called NGCs. NGCs act as a bridge between the proximal and distal ends of the injured nerve stump providing support for the infiltration of Schwann cells (SCs) which thereby guide the axonal regrowth [7, 8, 9, 10, 11, 12]. The ideal requirements of NGCs include biomimetic architecture, structural features to longitudinally align the regenerating axons, sufficient mechanical properties providing structural support, permeability to provide trophic support, conductivity, flexibility and biodegradability [13, 14]. Various biodegradable polymers like polycaprolactone, polylactic acid, alginate, chitosan etc., have been extensively studied in developing an ideal NGC [15, 16, 17, 18, 19].

Recent advances in the fabrication of nerve conduits have enabled in developing 3D bioprinted NGCs with features that closely mimic the nerve microenvironment. 3D bioprinting has various advantages, like (i) fabricating complex 3D structures with control over fiber alignment, porosity and surface topography, (ii) production of NGC with a combination of biomaterials, neurotrophic factors, and biomolecules, (iii) helps to closely mimic the nerve architecture and, (iv) allows for on‐demand, scalable fabrication of conduits [20, 21]. In particular, extrusion‐based bioprinting has been used extensively. It is a technique in which layer by layer deposition of biomaterial is carried out to print 3D structures. The advantages of extrusion‐based bioprinting include high accessibility, low cost and high accuracy [22]. In a study by Gao et al. [23], extrusion‐based bioprinting was used to develop a GelMA‐based four‐channel nerve conduit. The authors demonstrated that the bioprinted nerve conduit had adequate porosity, and stiffness comparable to nerve tissue. Further, the multi‐channel conduit increased the surface area enhancing the interaction of SCs like cells and axons. Although there have been numerous studies that have developed bioprinted nerve conduits, a fully robust system that can develop an ideal NGC is yet to be achieved.

To address this challenge, researchers have explored various biomaterials for fabricating NGC using extrusion‐based bioprinting. To this end, alginate has gained immense popularity. It is a negatively charged polysaccharide containing carboxyl groups on the ring structure of both β‐D mannuronate (M block) and α‐L guluronate (G) monomers. The presence of guluronic acid makes crosslinking of alginate with polyvalent ions such as Ca^2+^ easy under mild conditions. However, it lacks enough viscosity to be able to bioprint complex structures. To overcome the issue of viscosity, we blended MC with alginate and devised a dual crosslinking strategy to improve printability and preserve shape fidelity using the approach optimised and described in our previous work. Despite the promising results of our work, the alginate and MC nerve conduit was electrically insulating. To address this limitation while retaining the bioprinting advantages of our alginate/MC system, we explored the inclusion of an electroconductive material.

One of the important characteristic in an NGC is its electrical conductivity [24]. Neurons as well as SCs are highly responsive to electrical signals. Various studies have demonstrated that electrical stimulation enhances the movement of neuronal cells from the proximal to the distal stump in an appropriate pathway [25, 26, 27, 28, 29, 30]. Previously published literature have also shown that incorporation of electroconductive materials in NGC improved neuronal cell movement, neurite extension, neuro‐regeneration and enhances functional recovery [31, 32, 33]. To enhance the electrical characteristics of our Alg/MC nerve conduit, we hypothesised the incorporation of rGO within the polymeric matrix.

rGO is obtained by removing part of oxygen functional groups from graphene oxide. rGO is widely used in nerve tissue engineering due to its cost‐effectiveness, ease of synthesis, and good electrical conductivity when compared to pristine graphene and graphene oxide [34, 35, 36]. Zhang et al. [37], studied rGO/GelMA scaffolds in which 0.05% of rGO showed good mechanical properties, and promoted adhesion of bone marrow mesenchymal stem cells (BMSCs) and SCs. They also showed that the scaffolds containing both BMSCs and SCs achieved osteogenesis and neurogenesis within 2 months post subcutaneous implantation in rats. Although rGO has been extensively investigated within hydrogel systems and electrospun nerve conduits, its potential as a bioprinted conduit remains largely unexplored. To the best of our knowledge, 3D bioprinted nerve conduits fabricated using the blend of rGO within Alg/MC, along with dual crosslinking has not been previously investigated.

In this study, we present the first report of fabricating and characterizing an extrusion‐based 3D bioprinted nerve conduit using a blend of rGO within an Alg/MC hydrogel system. To improve the printability and shape fidelity of the bioprinted scaffold, a dual crosslinking approach (pre‐ and post‐ printing) with calcium chloride was developed. The printing parameters were optimized to obtain 10 mm‐long nerve conduits. The scaffolds were systematically evaluated for their morphological, physicochemical, and rheological properties. Cytocompatibility of the scaffolds was analysed using MTT and Live/dead assays. The electrical conductivity of the scaffolds was studied to confirm that the rGO concentration within Alg/MC/rGO scaffolds provides electrical cues. Additionally, the conduits were tested in vivo using a sciatic nerve injury model in Sprague Dawley rats. Functional recovery was assessed through the sciatic functional index. Tissue regeneration was evaluated through histological assessment. The in vivo results showed that the incorporation of rGO significantly promoted functional recovery.

## 2 Methodology

### 2.1 Characterization of rGO

rGO nanoparticles were purchased from Adnano Technologies (Cas no 7782‐42‐5)(India) and were thoroughly characterized.

#### 2.1.1 Scanning Electron Microscopy (SEM)

Scanning electron microscopy analysis was performed on rGO particles using Ultra55 FE‐SEM Carl Zeiss EDS (Carl Zeiss India Pvt. Ltd). A small quantity of rGO particles were mounted on a double‐ sided carbon adhesive tape followed by gold sputtering prior to imaging.

#### 2.1.2 Dynamic Light Scattering (DLS) and Zeta Potential Analyses

Dynamic light scattering and zeta potential analyses of the rGO particles were conducted using a Zeta‐PALS Analyzer (Brookhaven). The particles were dispersed in DI water prior to analysis, and their particle size was recorded.

#### 2.1.3 UV–Visible Spectroscopy

rGO particles were dispersed in DI water and spectral analysis was done using PerkinElmer Lambda 1050+ UV–Vis–NIR spectrometer (PerkinElmer).

#### 2.1.4 Fourier Transform Infrared (FTIR) Spectroscopy

FTIR spectroscopy of rGO particles was performed using a PerkinElmer Frontier instrument. The measurements were carried out from 400 to 4000 cm^−1^ at a wavenumber resolution of 4 cm^−1^.

#### 2.1.5 X‐ray Diffraction (XRD)

XRD analysis of rGO particles was performed using a Rigaku X‐ray diffractometer at a diffraction angle 2*θ* ranging from 10° to 90° at a rate of 2°/min using Cu Kα_1_ radiation on, operating at 40 kV and using 40 mA current. The d spacing was determined using the Bragg's equation.

### 2.2 Preparation of Alg/MC/rGO Hydrogel

**FIGURE 1:**
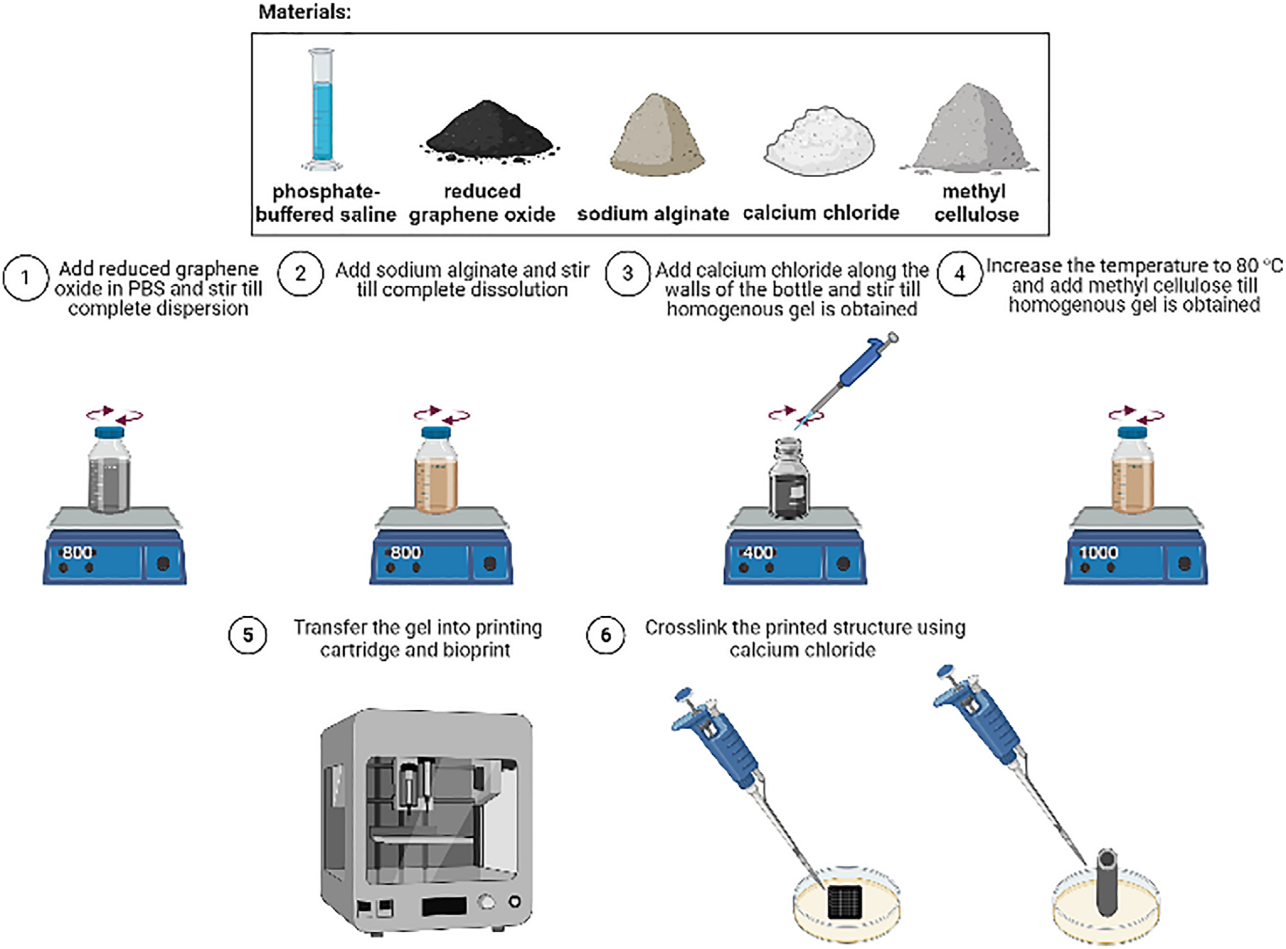
Schematic illustration of preparation of Alg/MC/rGO hydrogel and bioprinting.

A 0.5 mg/mL dispersion of rGO was prepared in 8 mL of PBS using a magnetic stirrer at 800 rpm and room temperature for 7 days. After dispersion, 500 mg of sodium alginate (SRL chemicals) was added and stirred at 800–1000 rpm at 45 °C till complete dissolution (∼2 h). Subsequently, calcium chloride (SRL chemicals, India) solution (36 mg in 2 mL PBS) was added and stirred at 400 rpm for 2 h. After complete dissolution, the temperature was raised to 80 °C, and 700 mg of MC (TCI) was added and stirred at 1000–1200 rpm for 3 h (Figure 1). The prepared hydrogel was gradually cooled to room temperature and stored at 4°C. The hydrogel was labelled as Alg/MC/rGO hydrogel.

Alg/MC hydrogel formulation was adopted from our previously published work, where the entire hydrogel composition optimisation procedure has been described. Briefly, 500 mg of sodium alginate was dissolved in 8 mL PBS and heated at 45 °C at 400 rpm. After complete dissolution, 36 mg of calcium chloride prepared in 2 mL of PBS was added till homogenous mixture is obtained. Then temperature was raised to 80 °C and 700 mg of MC was added and stirred at 1200 rpm. After complete dissolution, the temperature was reduced to 25 °C and stored at 4 °C.

### 2.3 3D Bioprinting and Rheological Analysis

Alg/MC/rGO hydrogel was subjected to bioprinting using an extrusion‐based Cellink Inkredible+ bioprinter with controllable pneumatic pressure. Bioprinting of Alg/MC/rGO hydrogel was optimized with various nozzle sizes and pressure to fabricate a grid and nerve conduit‐like structure on a 35 mm dish. Printing was carried out using a 20G nozzle at pressure ranging from 200–220 kPa. The bioprinted structures were crosslinked with 4% calcium chloride solution for 2–3 min.

The rheological behavior of Alg/MC, and Alg/MC/rGO hydrogels was assessed using an MCR302 rheometer (Anton Paar). A 25 mm parallel plate was used to measure the dynamic and loss moduli of the hydrogels at room temperature. Amplitude sweeps and shear thinning behavior were evaluated at a constant angular frequency of 10 rad/s.

### 2.4 Characterization of Alg/MC/rGO Scaffolds

#### 2.4.1 SEM Analysis

Scanning electron microscopy was performed on bioprinted Alg/MC/rGO scaffolds (grid and nerve conduit scaffolds) using Ultra55 FE‐SEM Carl Zeiss EDS (India). The lyophilized bioprinted scaffolds were placed on a double‐sided carbon adhesive tape, and gold sputter coating was done prior to imaging.

#### 2.4.2 Swelling Test

In vitro swelling behavior of the air‐dried bioprinted nerve conduits was evaluated by immersing pre‐weighed (w_i_) conduits in PBS and incubated at 37 °C. At various time intervals, the conduits were dabbed with tissue to remove excess PBS and weighed (w_f_) until equilibrium. The experiment was performed in triplicates and the swelling ratio was calculated using Equation (1).

(1) $$Swelling\;ratio\;\;\left(\%\right)=\left(\frac{\;w_{f}-\;w_{i}}{w_{i}}\right)\;\times 100$$

#### 2.4.3 Degradation Study

To assess the degradation behavior, air‐dried bioprinted nerve conduits were weighed (w_i_) and immersed in PBS and incubated at 37 °C. The weight loss was measured by removing the conduits from the PBS, blotting with tissue to remove excess PBS, and weighing (w_f_) at various time intervals. The experiment was carried out in triplicate. The degradation rate (%) was calculated using Equation (2).

(2) $$Degradation\;ratio\;\;\left(\%\right)=\left(\frac{w_{i}-w_{f}}{w_{i}}\right)\;\times 100$$

#### 2.4.4 FTIR

FTIR was performed on the lyophilised scaffolds (500 µl of the Alg/MC and Alg/MC/rGO hydrogels were placed in a 48‐well plate and crosslinked using 4% calcium chloride for 2–3 min) using a PerkinElmer Frontier instrument to study the presence of functional groups. The measurements were carried out from 400 to 4000 cm^−1^ at a wave number resolution of 4 cm^−1^ and 16 scans per sample were averaged and presented.

#### 2.4.5 Conductivity Test

The air‐dried Alg/MC and Alg/MC/rGO scaffolds in triplicates were subjected to impedance analysis using an impedance analyzer (Hioki LCR Impedance analyzer IM3570, Japan). The dry scaffolds were connected to the impedance analyser using titanium electrodes. The AC generator provided a constant current from high to low frequency. At each frequency, impedance parameters including magnitude, real and imaginary components and phase angle were recorded. Measurements were taken across a fixed voltage of 0.1 V and a frequency range of 10–5 000 000 Hz. Bulk resistance values were extracted from Nyquist plots. Effective conductivity was calculated using Equation (3)

(3) $$Conductivity=\frac{Thickness}{Resistance\times \;area}\;$$

#### 2.4.6 Cytocompatibility Tests

To evaluate cell viability, live/dead staining (Invitrogen, USA) was performed. The lyophilized Alg/MC and Alg/MC/rGO scaffolds were placed in rat Schwann cell (RSC96) culture media and incubated in a humidified incubator at 5% CO_2_, and 37 °C. The conditioned media were collected at day 1, 5, 7, and 14.RSC96 cells (CRL‐2765 ATCC, USA) were seeded on a 96‐well plate at a concentration of 1000 cells/well in RSC96 cells culture media consisting of DMEM, 10% fetal bovine serum (FBS), penicillin‐streptomycin and antibiotic‐antimycotic (Gibco, India). After 24 h of culture in a humidified incubator at 5% CO_2_ and 37 °C, the culture media was removed and replaced with 100 µL of the conditioned media and incubated for 24 h. The media was discarded, and 100 µL of calcein AM and ethidium homodimer (1:4) in PBS was added and incubated for 40 min in the dark at in a humidified incubator at 5% CO_2_ and 37 °C. The cells were imaged under fluorescence microscopy (Nikon) and analysed using ImageJ software.

The indirect method of MTT (3‐(4,5‐dimethyl‐2‐ thiazolyl)‐2,5‐diphenyl‐2H‐tetrazoliumbromide) assay (HiMedia) was done to study cell proliferation [38, 39]. The lyophilized Alg/MC and Alg/MC/rGO scaffolds were placed in RSC96 cells culture media and incubated in a humidified incubator at 5% CO_2_, and 37 °C. The conditioned media was collected at day 1, 5, 7, and 14. RSC96 cells were seeded on a 96‐well plate at a concentration of 1000 cells/well in RSC96 cells culture media. After 24 h of culture in a humidified incubator at 5% CO_2_ and 37 °C, the culture media was removed and replaced with 100 µL of the conditioned media and incubated for 24 h. Post incubation, 200 µL of MTT reagent (0.5 mg/ml) was added to each well and incubated for 4 h in a humidified incubator at 5% CO_2_ and 37 °C in dark. After 4 h incubation, 100 µL DMSO was added and incubated on a shaker at room temperature for 30 min. The absorbance was measured at 570 nm using a multiplate reader (HH34000000, Perkin Elmer (Ensight)). Cell viability was calculated using Equation (4), where untreated cells were taken as the control. The graphs were plotted using GraphPad Prism software.

(4) $$Cell\;viability\;\%=\left(OD\;sample/OD\;control\right)\;\times 100$$

### 2.5 In Vivo Sciatic Nerve Injury Model Analysis

#### 2.5.1 Animal Surgery and Scaffold Implantation

Female Sprague‐Dawley (SD) rats (250–300 g) were purchased and housed in specified‐pathogens free facility of Animal House and all procedures were performed in accordance with the Institutional Animal Ethical Committee (IAEC) approval (NCOPS/IAEC/04/25‐56) and were conducted in accordance with the guidelines of the National Research Council's Guide for the Care and Use of Laboratory Animals, as well as the Committee for Control and Supervision of Experiments on Animals (India). Female rats were selected to reduce aggressive behavior and minimize fighting‐related injuries during post‐operative care. The experimental procedures were performed under ketamine anaesthesia (70 mg/kg body weight). After shaving the hair at the surgical site, an incision was made in the skin and the subcutaneous muscle layer to expose the sciatic nerve. Three rats were equally divided into three groups, control group (without injury), the injury group and Alg/MC/rGO nerve conduit group. In the injury group, left limbs underwent sciatic nerve transection of 5 mm and were placed over the muscle without approximation or suturing. In the nerve conduit group, 5 mm of sciatic nerve was resected on the left limb and Alg/MC/rGO bioprinted nerve conduit was placed and glued using a skin adhesive (Liquiband Optima). Rats were euthanized using high dose of thiopentone anaesthesia at 21 days post implantation.

#### 2.5.2 Evaluation of Sciatic Nerve Functional Recovery

Functional analysis of axonal regeneration after implantation was evaluated using the sciatic function index (SFI). The rats were trained to walk along a wooden walkway to establish a steady and natural walking pattern to get used to the setup. For the gait analysis, a sheet of white paper is laid out along the walkway, and the hind paws are coated with water‐soluble paint at days 14 and 21 post implantation. As the rats walk, the paw prints were recorded on the paper for both the injured (experimental) and non‐injured (control) hind limbs. The SFI was calculated using the Bain–Mackinnon–Hunter Equation (5) [40],

(5) $$\begin{array}{ccc} SFI & = & -38.3\times \frac{EPL-NPL}{NPL}+109.5\times \frac{ETS-NTS}{NTS}\\ & & +\;13.3\times \frac{EIT-NIT}{NIT}-8.8 \end{array}$$

where, EPL: experimental print length, NPL: normal print length, ETS: experimental toe spread (first to fifth toe), NTS: normal toe spread, EIT: experimental intermediate toe spread (second to fourth toe) and NIT: normal intermediate toe spread.

#### 2.5.3 Histological Analysis of Nerves

Animals were sacrificed on day 21, nerve stumps were harvested and fixed in 10% formalin to preserve the tissue section. Tissue or nerve from the proximal and distal parts of the resected portion of the nerve was excised. The tissues were embedded in paraffin and sectioned longitudinally for histological analysis. Histological assessment included hematoxylin and eosin (H&E) staining (AMD Labs) to evaluate the tissue architecture and cellular changes within the harvested tissue. Luxol fast blue stain (HiMedia) was then carried out to stain the lipoproteins present on the surface of the myelin sheath. Further, Masson's trichrome staining (AMD Labs) as per the manufacturer's instructions was done to evaluate the presence of collagen. The images were observed under a phase contrast microscope (Nikon 80i) and the images were analysed using ImageJ software and color intensity was measured and plotted using GraphPad Prism software.

### 2.6 Statistical Analysis

All quantitative data are presented as mean ± standard deviation (SD). The results were analyzed using two tailed unpaired t‐test, two‐way analysis of variance (ANOVA), followed by Sidak's multiple comparison test, and Ordinary one‐way analysis of variance followed by Tukey's multiple comparisons test. Statistical analysis was conducted with at least 3 independent samples per experiment (*n* = 3). A p<0.05 was considered statistically significant.

## 3 Results and Discussion

**FIGURE 2:**
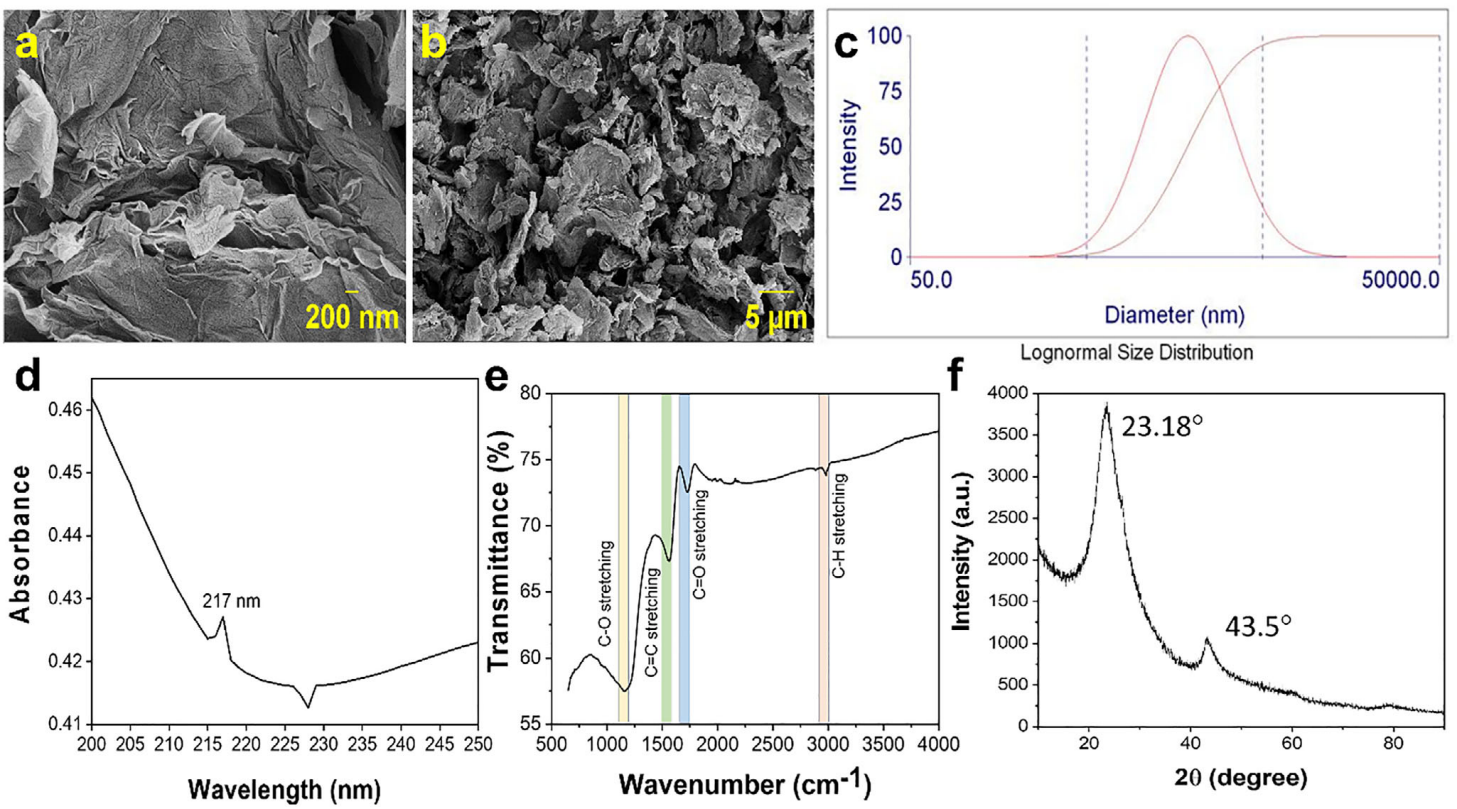
Representative scanning electron microscopy images of rGO at (a) 50000x magnification, (b) 5000x magnification, (c) Dynamic light scattering analysis, (d) UV–vis spectroscopy analysis, (e) FTIR analysis and (f) x‐ray diffraction analysis of rGO.

Among different conductive nanofillers, rGO has high electrical conductivity, and a good biocompatibility profile [41, 42, 43]. A commercial rGO was used in this study, which was thoroughly characterised prior to use. The morphological characteristics of rGO were examined using scanning electron microscopy as shown in Figure 2a,b. A wrinkled, folded, and crumbled morphology was observed for rGO sheets, which was in line with previously published literature [41, 44]. At lower magnification (Fig 2b), stacked features were observed, potentially caused by van der Waals interactions between rGO sheets [45]. Dynamic light scattering analysis (DLS) was conducted to assess the hydrodynamic diameter and size distribution of rGO in aqueous dispersion. DLS revealed a multimodal size distribution with an average hydrodynamic radius of ∼200 nm and PDI of 0.379 (Figure 2c). The partially stacked structure could explain the broad distribution observed under the DLS. Zeta potential was analysed to determine the surface charge and stability of rGO in suspension. The zeta potential of −40.57 mV was obtained, indicating negative surface charge and good colloidal stability in suspension. The zeta potential value of >30 mV is indicative of strong colloidal stability. The UV–Vis absorption spectrum is presented in Figure 2d, showing an absorption peak at ∼217 nm, which corresponds to the π‐π* electronic transitions of C═C in graphitic domains. The presence of ∼217 nm peak corresponds to sp^2^ hybridized carbon structure [46]. FTIR was used to characterize the presence of different functional groups present in rGO. FTIR analysis revealed peaks at 2981 cm^−1^ for C─H stretching, 1728 cm^−1^ for C═O carbonyl stretching, 1558 cm^−1^ for stretching of C═C alkene group and 1155 cm^−1^ for C─O epoxide group stretching. We observed presence of no characteristic oxygen‐containing functional groups (e.g. ‐OH) in the FTIR analysis. The obtained peaks are indicative of rGO, which is in line with previous literature [47, 48, 49, 50]. XRD analysis revealed rGO characteristic peaks at 23.18° and 43.5° [50]. The d‐spacing of 0.38 nm was obtained using the 23.18° peak. The results obtained were in concord with the results reported previously in the literature [47, 51, 52].

Alginate is one of the most abundantly used natural polymers in 3D bioprinting owing to its properties like biocompatibility, and ability to undergo ionic crosslinking. However, pristine alginate hydrogels have poor mechanical stability, low viscosity, and lack shape fidelity post‐printing. In our previous study, calcium chloride was added to alginate as a pre‐print crosslinker to improve the viscosity. However, satisfactory printing was not obtained. Therefore, MC was incorporated into the alginate and calcium chloride blend to improve printability. The obtained alginate, calcium chloride and MC hydrogel (Alg/MC hydrogel) enabled successful bioprinting of a nerve conduit‐like structure with dimension 10 mm length and 5 mm diameter. However, despite obtaining a good printed nerve conduit‐like structure, the printed conduit exhibited limited shape fidelity post‐printing. To address this limitation, post‐print crosslinking using calcium chloride was subsequently developed and reported by us in our previous work. Post‐print crosslinking improved the structural stability of the bioprinted Alg/MC nerve conduit. However, an effective nerve conduit must also provide electrical cues as a peripheral nerve is inherently electrically conductive, which was lacking in Alg/MC nerve conduit. Based on this, we hypothesised that the incorporation of rGO into Alg/MC hydrogel would impart electrical conductivity into the bioprinted nerve conduit.

rGO was initially dispersed in PBS and alginate was added to obtain a homogeneous mixture. rGO not only provides electrical conductivity but also acts as a nanofiller for the hydrogel network due to its large surface area and presence of oxygen containing functional groups. The rGO sheets undergo interfacial interactions with hydroxyl and carboxyl groups of the alginate by H‐bonding [53]. This interaction leads to the formation of interpenetrating networks between alginate and rGO. The addition of calcium chloride to the alginate and rGO blend leads to the formation of ionic bonds between carboxylic acid groups of alginate and calcium cations of the calcium chloride initiating a crosslinking process. The concentration of calcium chloride added to alginate was optimized in our previous work to obtain adequate viscosity for printing. To further improve the printability, MC was added to the alginate and rGO blend. The crosslinking between alginate, rGO and MC can be attributed to interpenetration of MC and alginate network and hydrophobic interaction within the MC molecules [54]. Bioprinting of grid and nerve conduit was carried put under optimized printing conditions as mentioned in Section 2.3.

**FIGURE 3:**
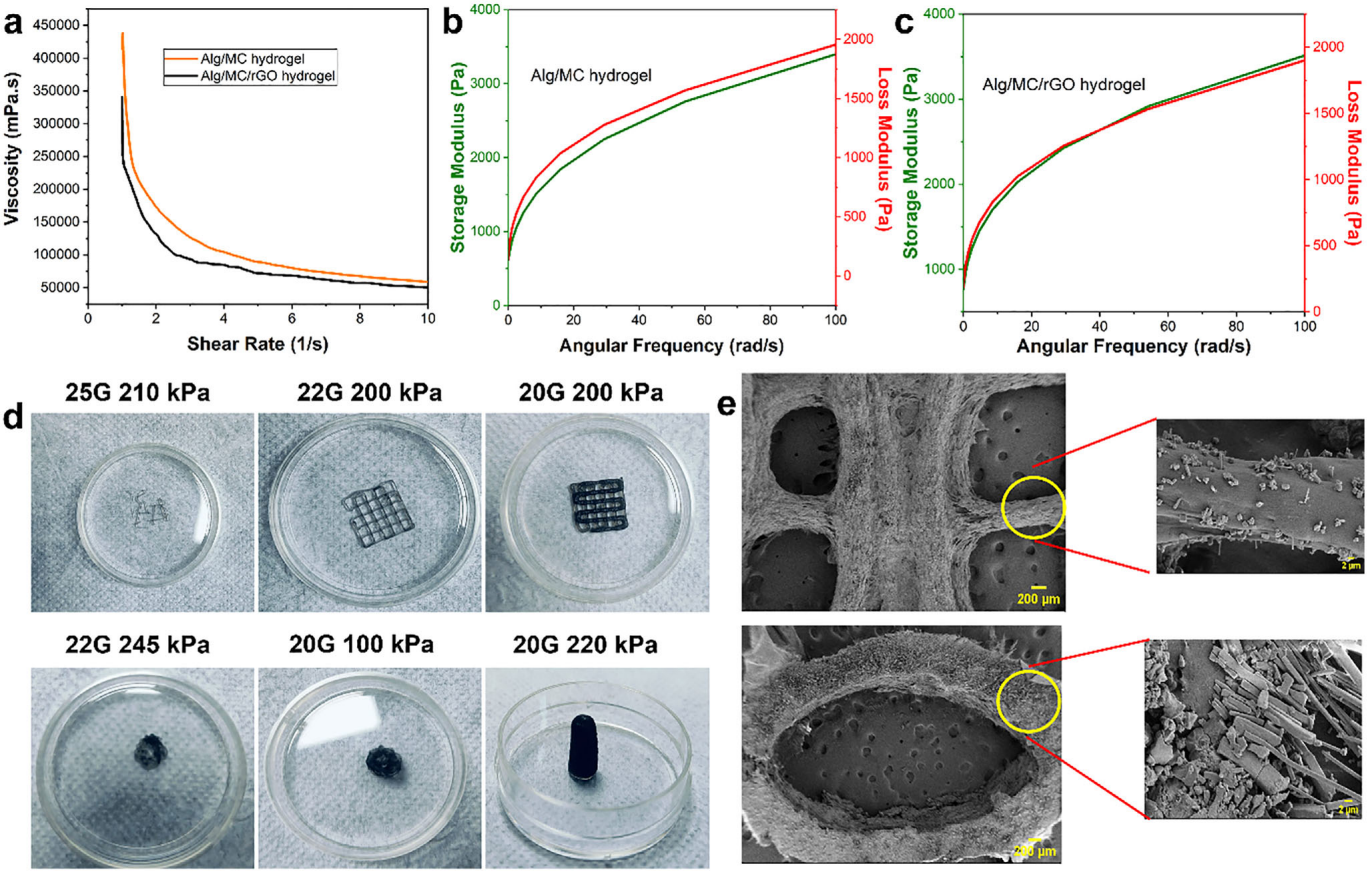
Rheological analysis (a) Viscosity vs shear rate at 37°C, and Storage modulus, loss modulus vs angular frequency of (b) Alg/MC hydrogel, (c) Alg/MC/rGO hydrogel, (d) Representative macroscopic images of 3D bioprinted Alg/MC/rGO structures and (e) Representative scanning electron microscopic images of 3D bioprinted Alg/MC/rGO structures: Top panel bioprinted grid like structure at 69x and 5000x  magnification, Bottom panel bioprinted nerve conduit at 61x and 5000x magnification. Scale bar = 200 µm and 2 µm.

Successful extrusion of hydrogel during bioprinting is highly dependent upon its rheological properties. Rheological properties such as viscosity, storage modulus (G′), and loss modulus (G‴) must be characterized to understand the elastic properties of the hydrogel. The rheological properties of Alg/MC and Alg/MC/rGO hydrogels are presented in Figure 3. As seen in Figure 3a, viscosity was measured as a function of shear rate. Both hydrogels demonstrated typical shear‐thinning property, where the viscosity decreases with an increase in shear rate. At low shear rates (0‐1 s^−1^), the viscosity of both hydrogels was > 250 000 mPa s indicating a dense polymeric network. As shear rate increases, viscosity is reduced to 50 000 mPa s (6–8 s^−1^) and plateauing beyond 8 s^−1^. It is also observed that at any given shear rate, Alg/MC/rGO exhibited lower viscosity when compared to Alg/MC hydrogel. Further, an amplitude sweep test was carried out to assess the storage and loss modulus as a function of angular frequency. The hydrogel used for bioprinting should demonstrate viscoelastic behaviour i.e., it should show liquid‐like behaviour to undergo extrusion from the bioprinting nozzle and prevent its clogging, and it should also demonstrate elastic‐like behaviour to maintain the structural integrity of the printed scaffolds. The storage modulus of a hydrogel is a measure of energy within the hydrogel during shearing and depicts the elastic‐like nature of the hydrogel. The loss modulus represents the energy dissipated from the hydrogel system during the shearing process, depicting its liquid‐like nature. During bioprinting, the storage modulus should be greater than the loss modulus but not be too high, as this may prevent its extrusion from the nozzle. Therefore, there should be adequate balance between the storage and loss modulus [55, 56, 57]. In our study, we observed that the storage modulus of Alg/MC hydrogel was lower than the loss modulus (G′<G″), indicating that the hydrogel shows liquid like behavior as seen in Figure 3b. It was also observed that there was no cross over between G′ and G″ indicating that the hydrogel showed similar behaviour through‐out the measured angular frequency range (0–100 rad/s). On the other hand, Alg/MC/rGO hydrogel showed slight increase in G″ when compared to G′ (G′<G″). However, this difference between G″ and G′ was very small indicating liquid like behaviour of the hydrogel. The crossover of G″ and G′ was observed between 40–50 rad/s, indicating that Alg/MC/rGO hydrogel showed a shift from liquid to elastic nature. This was confirmed when printing was carried out, where we observed that the Alg/MC/rGO bioprinted structures showed visible shape fidelity in comparison to Alg/MC bioprinted structures.

The Alg/MC/rGO bioprinted structures (grid and nerve conduit) were subjected to scanning electron microscopy to understand its surface morphology. We observed in Figure 3e (top panel) that the grid‐like bioprinted scaffold showed well defined, uniform strand deposition with interconnected pores, demonstrating good shape fidelity of the bioprinted structure. When observed in 5000x magnification, flake‐like structures were observed on the surface which increases the surface roughness to enhance cell attachment [58]. The bottom panel in Figure 3e shows scanning electron microscopic images of a bioprinted nerve conduit. The conduit exhibited a smooth and continuous boundary or wall with a clearly defined inner lumen. In the 5000x magnification image of the nerve conduit, flake‐like rGO structures were seen to be embedded within the scaffold like the grid‐like structure. indicating that the rGO was uniformly dispersed in the Alg/MC/rGO hydrogel.

The lyophilised scaffolds were subjected to FTIR analysis to potentially understand the chemical interactions within the scaffolds. The Alg/MC and Alg/MC/rGO lyophilised scaffolds exhibited characteristic peaks of alginate and MC. In Alg/MC scaffold, a broad band around 3300 cm^−1^ corresponding to O─H stretching vibrations was observed, indicating hydroxyl groups of alginate and MC. The peak at 2900 cm^−1^ corresponds to C─H stretching and a sharp band, and 1700 cm^−1^ corresponds to C═O stretching of alginate carboxyl group [59, 60]. The peaks at 1000–1100 cm^−1^ are related to C─O─C and C─O stretching vibration of the polysaccharide backbone [61, 62]. In Alg/MC/rGO scaffolds the intensity of the peaks was reduced when compared to Alg/MC scaffolds. Reduced intensity of peaks at ∼3300, ∼1700, and ∼1000 cm^−1^ could be induced by the introduction of rGO in the scaffold, which might be suppressing the signal under the FTIR.

**FIGURE 4:**
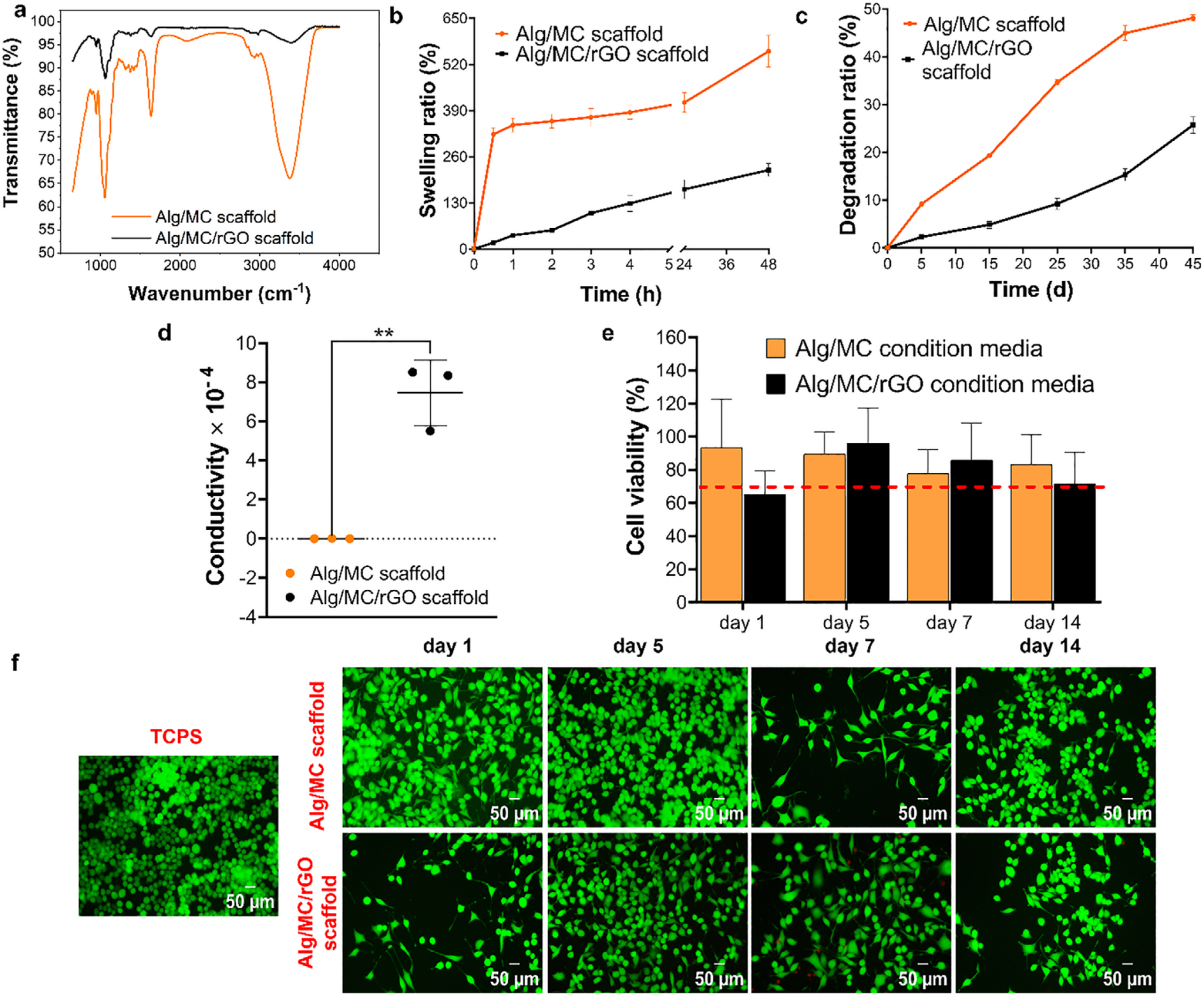
(a) FTIR analysis, (b) Swelling test, (c) Degradation test and (d) Conductivity test (e) MTT assay and (f) Representative fluorescence images of RSC96 cells from live/dead assay. Statistical analysis was done using the two tailed unpaired t‐test with ^**^: *p*< 0.01. Data are presented as average ± standard deviation (*n* = 3).

The swelling behavior of Alg/MC and Alg/MC/rGO scaffolds in PBS is shown in Figure 4b. The Alg/MC scaffold achieved swelling equilibrium at 30 min, whereas the Alg/MC/rGO scaffolds did not attain equilibrium even at 48 h. Alg/MC/rGO scaffolds demonstrated a linear increase in swelling ratio. Alg/MC scaffold showed an increased swelling ratio when compared to Alg/MC/rGO scaffolds. Alg/MC scaffolds reached a swelling ratio of 348.93% ± 16.13% in 1 h and continued to increase to 557.38% ± 36.21% at 48 h. This rapid swelling can be due to the hydrophilic nature of both alginate and MC. In the case of Ag/MC/rGO scaffolds, swelling at 1 h was 38.38% ± 4.16%, which increased only to 221.97% ± 14.73% at 48 h, indicating relatively lower swelling ability compared to Alg/MC scaffolds. The swelling ability of <400 wt.% is considered as low swelling ability, whereas >1000 wt.% indicates high swelling ability [63]. The reduced swelling in Alg/MC/rGO scaffolds compared to Alg/MC scaffolds is hypothesised to be caused by the rGO, which reduced the free volume available for water diffusion in the scaffold, influencing their (scaffold) swelling behaviour [64, 65]. The interaction between alginate, MC, and rGO potentially produced a highly crosslinked polymer network which could explain the reduced water absorption and retention of structural stability of the Alg/MC/rGO scaffolds. Retention of structural stability is important for the printed structures to preserve their geometry and shape fidelity. The swelling of the scaffolds is important as this enables the transport of nutrients and waste removal from the scaffolds. Swelling also maintains the softness of a hydrogel and helps to keep cells hydrated, promoting cell adhesion and proliferation [66].

Figure 4c shows the degradation ratio of the scaffolds. Degradation studies were conducted keeping day 5 as the initial time point as degradation was only observed post day 5. Alg/MC scaffolds exhibited rapid degradation with ∼50% weight loss, while Alg/MC/rGO scaffolds exhibited ∼25% weight loss during a 45‐day study. This reduced degradation rate of scaffolds containing rGO corroborates the hypothesis that rGO containing scaffold had greater and potentially stronger crosslinking than Alg/MC scaffolds. Further, the hydrophobicity of rGO could also inhibit the inflow of PBS into the hydrogel network [67]. We assume that this reduced degradation in Alg/MC/rGO scaffolds will make the bioprinted nerve conduit stable for a long time while providing structural cues for the SCs and aids in regeneration.

Next, we explored the electrical properties of the produced scaffolds using an impedance analyzer. The Nyquist plots were analyzed to determine the electrical conductivity of scaffolds. As seen in Figure 4d, conductivity values for Alg/MC scaffolds were 7.6 × 10^−5^ ± 6.5 × 10^−5^ S/m and that of Alg/MC/rGO scaffolds was 7.5 × 10^−2^ ± 1.4 × 10^−2^ S/m. As expected, Alg/MC scaffolds showed negligible conductivity. The electrical conductivity is an important intrinsic property previously reported to improve neuronal cell activity [68, 69]. Notably, the electrical conductivity range of human nerve is 8.0 × 10^−2^ to 1.3 S/m, indicating that Alg/MC/rGO scaffolds exhibited conductivity values within the physiologically relevant range [24, 70].

The in vitro cytocompatibility of Alg/MC and Alg/MC/rGO scaffolds were assessed using the MTT and live/dead assays on RSC96 cells. The MTT assay was carried out at 24 h after obtaining the conditioned media at four time points (1, 5, 7, and 14 days). The dotted (red) line in Figure 4e at 70% represents the cytotoxicity threshold above which materials are considered to be biocompatible in accordance to the current ISO 10993–5:2009 (E) standard [71]. It was observed that the conditioned media collected at 5, 7, and 14 days showed no cytotoxic effect. However, day 1 conditioned media from Alg/MC/rGO scaffolds showed a slight reduction in cell viability (∼65%) when compared to days 5,7, and 14. As seen in live‐dead staining (Figure 4f), cellular viability was maintained through day 1, 5, 7, and 14 in all scaffold groups. The predominance of green fluorescence (live cells) was observed across both Alg/MC and Alg/MC/rGO groups at days 1, 5, 7, and 14. Cells exposed to conditioned media from both Alg/MC and Alg/MC/rGO scaffolds at all time points (1, 5, 7, and 14 days) exhibited a normal spindle‐shaped morphology and good cell spreading. Few dead cells were observed in cells exposed to Alg/MC/rGO conditioned media at day 7. Overall, the live/dead assay corroborated MTT assay findings and confirmed that conditioned media obtained from both Alg/MC and Alg/MC/rGO scaffolds supported sustained cell viability up to 14 days.

Owing to the biocompatibility, and conductive properties of the Alg/MC/rGO scaffolds (lyophilised), we studied the neural regeneration potential of the 3D bioprinted nerve conduits in vivo in a well‐established 5 mm sciatic nerve defect Sprague‐Dawley rat model and monitored them for 21 days. The animals were randomly divided into two groups: injury control (no treatment at injury site) and Alg/MC/rGO conduit treatment group. Functional recovery was monitored using behavioural assessment and regenerative outcome was studied through histological assessment. All experiments and handling of animals was performed following CCSEA (Committee for Control and Supervision of Experiments on Animals) guidelines. The functional assessment such as the sciatic functional index was performed at two‐time intervals (day 14 and 21) after which animals were sacrificed to assess the extent of nerve regeneration.

**FIGURE 5:**
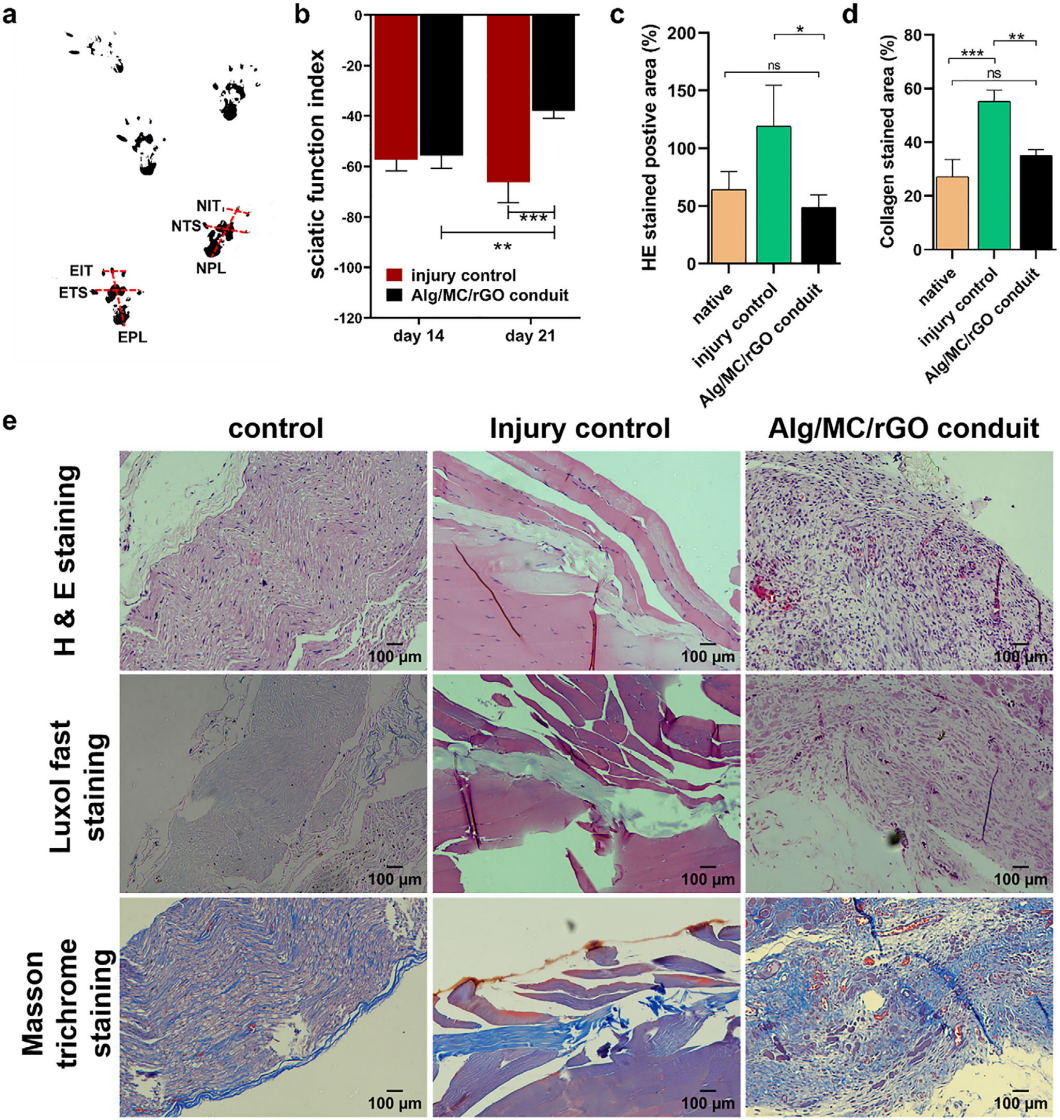
(a) Digital image of footprint of control uninjured animal, Analysis of nerve regeneration potential of conduit through histological analysis (b) Quantitative estimation of SFI of different groups at day 14 and 21. (c) Quantification of H&E‐stained positive area. (d) Quantification of Collagen‐stained area. (e) Bright‐field images of H&E, Luxol fast, and Masson's Trichrome staining. Scale bar = 100 µm. Data re represented as mean ± SD (*n* = 3). ^***^ denotes *p* < 0.001, ^**^ denotes *p* < 0.01, ^*^ denotes *p* < 0.05, ns denotes not significant.

Sciatic function index (SFI) measures the level of motor activity of the sciatic nerve which was calculated by comparing the print of the damaged hind paw of the injured leg to the corresponding contralateral paw in all animals at day 14 and 21. SFI values were calculated using the standard Bain–Mackinnon–Hunter formula. It is well established that SFI values range from ∼0 (normal nerve function) to −100 (complete dysfunction), and thus the native group is considered to have an SFI value close to 0. The native group represents baseline function and therefor is not included in the graphical representation [72]. It was observed (Figure 5b) that the SFI values for injury control (−57.3 ± 3.6) and Alg/MC/rGO (−55.6 ± 4.1) conduit treatment group were similar on day 14 indicating that the animals showed impairment in walking. However, on day 21, between the two groups, Alg/MC/rGO conduit treatment group (−37.9 ± 2.4) exhibited a significant increase in SFI when compared to injury control (−66.2 ± 6.5). It was also observed that there was a significant increase (*p* < 0.01) in SFI in Alg/MC/rGO conduit treatment group when compared from day 14 to 21. This enhanced functional recovery can be attributed to the presence of the conductive rGO nanofiller in the conduit. However, it is also necessary to consider that the nerve gap of 5 mm was done in our study. Recent studies have demonstrated that rGO‐based silicon conduits promoted the behaviour of nerve cells as well as influence the neurogenic differentiation of stem cells [73]. Additionally, it is also reported in the literature that freeze‐dried alginate gel covered by polyglycolic acid mesh was able to successfully regenerate a 50 mm sciatic nerve defect in cats [74]. In another study by Szarek et al. [75], nerve conduits were fabricated using a polyurethane/polylactide blend and calcium alginate fibers were filled in lumen of the conduits. It was observed and concluded that calcium alginate fibers created a favorable microenvironment for outgrowing nerves in a 10 mm rat sciatic nerve injury. However, these studies evaluated alginate or rGO in isolation and employed conventional fabrication techniques. In the present study, we integrated alginate, MC and rGO to impart both conductive and neuro‐inductive properties to create a more supportive microenvironment for axonal regeneration.

Next, a histological investigation of the native control, injury control and the conduit treatment group were carried out to determine inflammatory responses and regenerative outcome. Longitudinal sections of the regenerated nerve tissue from the injured site were stained with H&E, Luxol fast and MTS staining 21 days post‐surgery. The middle part of the injured nerve covered with conduit was taken for the analysis. H&E staining showed the presence of normal nerve architecture in control sections (no injury). In contrast, the injury control exhibited severe disruption in nerve structure. In Alg/MC/rGO conduit group an organised nerve structure was observed with infiltration of greater number of cells like SCs, inflammatory cells (Fig 5e). Quantification of the H&E‐stained positive area was done using Image J software by measuring the percentage area of stained regions using colour deconvolution plugin. The area of each stain was measured and added to plot the graph. It was observed that, in injury control group (119.2% ± 28.7%) had a significantly higher (*p* < 0.05) stained area when compared to Alg/MC/rGO conduit group (48.7% ± 8.9%) and native nerve (64.1% ± 12.7%) indicating the presence of dense fibrous connective tissue formed after injury. The Alg/MC/rGO conduit group showed similar intensity when compared to native nerve (Figure 5c). There was an increase in the presence of immune cells and SCs when compared to native nerve and injury control. The presence of cells even after 21 days of injury may be attributed to Wallerian degeneration. The implanted nerve conduit promotes recruitment of SCs and macrophages at the injury site to clear the debris. Post 14 days of injury, the macrophages release anti‐inflammatory cytokines and repair SCs guide the regrowing axons [6]. In a study by Ghosh et al. [76], the presence of nerve cells were observed in H&E stained nerve sections even as late as 8 weeks post treatment in vivo.

To assess the myelin presence and distribution, Luxol fast blue staining was carried out. Myelin is an insulating protective layer around nerve fibers that support axonal function and accelerates nerve signalling conduction. This stain mainly stains the phospholipids, which are the primary constituent of myelin. As seen in Figure 5e, a well‐preserved nerve architecture with uniform staining of the intact myelin fibers was observed in the native control group. In contrast, patchy blue stain was observed in the injury control indicating myelin loss and degenerative changes. However, blue stain was observed throughout the nerve architecture in Alg/MC/rGO conduit group indicating partial restoration of myelination.

The Masson's trichrome staining was conducted to evaluate the extent of injury and deposition of collagen. In native control, normal nerve morphology with minimal collagen deposition (blue) was observed. In injury control, severe disruption of nerve fibers and abundant collagen accumulation was observed indicating scar formation and fibrosis. This is consistent with post nerve injury response in which scar tissue formation is observed [77, 78]. In the case of Alg/MC/rGO conduit group, collagen deposition was markedly reduced compared to the injury control, indicating that the conduit provided a more conducive microenvironment for axonal regeneration. On quantification (Fig 5d), it was observed that there was a significant increase (*p* < 0.001) in fibrous tissue formation in the injury control (55.2% ± 3.4%) when compared to the native control group (27.1% ± 5.2%) as anticipated. In contrast, Alg/MC/rGO nerve conduit group (35.0% ± 1.8%) showed a significant reduction (< 0.01) in the presence of fibrous tissue when compared to the injury control group. It was also observed that there was a marginal increase in collagen deposition in Alg/MC/rGO nerve conduit group when compared to the native nerve which was however not significant. Previous studies have shown that alginate creates a microenvironment that promotes SCs proliferation and migration while inhibiting fibroblast‐mediated scar tissue formation [79, 80, 81]. Overall, the histological results and sciatic functional analysis suggest that Alg/MC/rGO nerve conduit aided in successful nerve regeneration. While this study demonstrates promising biocompatibility and functional recovery, it represents an initial proof‐of‐concept with a short‐term (21‐day) evaluation in a small defect model. Conductive biomaterials are known to promote neurite outgrowth and nerve regeneration through electrical cues [82, 83]. Limitations of our study includes lack of long‐term studies, advanced control groups, direct neurite analysis, and mechanical characterization. Future work will focus on larger defect models, extended evaluation, detailed toxicity profiling, and incorporation of biological cues. These efforts are essential to advance the conduit toward clinical translation.

## 4 Conclusion

This study demonstrates the fabrication and evaluation of a 3D bioprinted nerve conduit based on Alg/MC/rGO hydrogel using extrusion based bioprinting. rGO was thoroughly characterized to understand its properties. The optimized hydrogel exhibited shear‐thinning behavior required for extrusion‐based bioprinting. A unique dual crosslinking strategy using calcium chloride was devised to obtain good printability and shape fidelity. Physicochemical analysed confirmed uniform distribution of rGO within the Alg/MC blend and conductivity of the scaffolds were found to be within the physiological range of peripheral nerves. In vitro cytocompatibility assays verified that the Alg/MC/rGO scaffolds supported RSC96 cells viability above ISO‐defined cytocompatibility thresholds. Further, in vivo implantation in a 5 mm sciatic nerve transection model in SD rats showed marked improvement in functional recovery as evident by better sciatic functional index at 21‐day post implantation. Histological evaluation confirmed reduced fibrosis, better myelination, and well‐aligned regenerating nerve fibers. Overall, we could conclude that 3D bioprinted Alg/MC/rGO nerve conduit along with the dual crosslinking strategy shows great promise as an advanced 3D bioprinted nerve conduit platform for peripheral nerve regeneration.

## Conflicts of Interest

The authors declare no conflicts of interest.

## Supporting information


**Supporting File**: mabi70223‐sup‐0001‐SuppMat.docx.

## Data Availability

The data that support the findings of this study are available from the corresponding author upon reasonable request.
