# Supplementary material for: In Vivo Evaluation of Conductive Biopolymer‐Based 3D Bioprinted Nerve Conduit in Sciatic Nerve Injury Repair
Source: Macromol Biosci. 2026 Jul 18;26(7):e70223. doi: 10.1002/mabi.70223 (PMC13380635; doi:10.1002/mabi.70223)
Supplement: Supplementary file 1 — Supporting File: mabi70223‐sup‐0001‐SuppMat.docx. [file MABI-26-e70223-s001.docx]

In Vivo Evaluation of Conductive Biopolymer-based 3D Bioprinted Nerve Conduit in Sciatic Nerve Injury Repair

Nasera Rizwana,^a^ Yogesh H S,^b^ Kaustubh Raundal,^a^ Janani Sriramakrishnan,^c^ Shounak De,^c^ Syed Sahal,^b^ Goutam Thakur,^c^ Ashwath Acharya,^d^ Vipul Agarwal,^e*^ Manasa Nune^a*^

*^a^Manipal Institute of Regenerative Medicine, Manipal Academy of Higher Education, Manipal 576104, Karnataka, India*

*^b^Department of Pharmacology, NITTE College of Pharmaceutical Sciences (NITTE Deemed to be University), Bangalore Campus, Karnataka, India*

*^c^Manipal Institute of Technology, Manipal Academy of Higher Education, Manipal 576104, Karnataka, India*

*^d^Department of Hand Surgery, Kasturba Medical College, Manipal Academy of Higher Education, Manipal 576104, Karnataka, India*

*^e^Department of Materials Science and Engineering, Monash University, Clayton, VIC, 3800 Australia*

*Corresponding authors: [agarwalvipul84@gmail.com](mailto:agarwalvipul84@gmail.com); [manasa.nune@manipal.edu](mailto:manasa.nune@manipal.edu)

**Supporting Information**

**Optimization of Bioprinting of Alg/MC hydrogel**


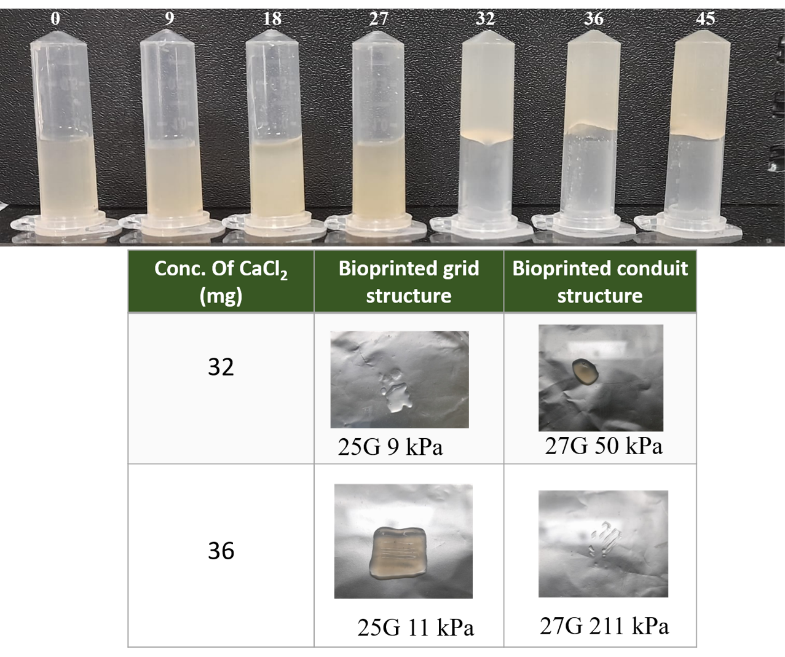


**Figure S1:** Optimization of bioprinting of alginate (top panel) non-flow behavior of various concentrations of calcium chloride in 1 gm alginate. Numbers on eppendorf tubes are 0, 9, 18, 27, 32, 36, 45 mg. (bottom panel) optimization of bioprinting of alginate with 32 and 36 mg of calcium chloride. At 36 mg calcium chloride, 25G nozzle size and 11 kPa pressure, a proper grid structure was printed and conduit structure was not printed.


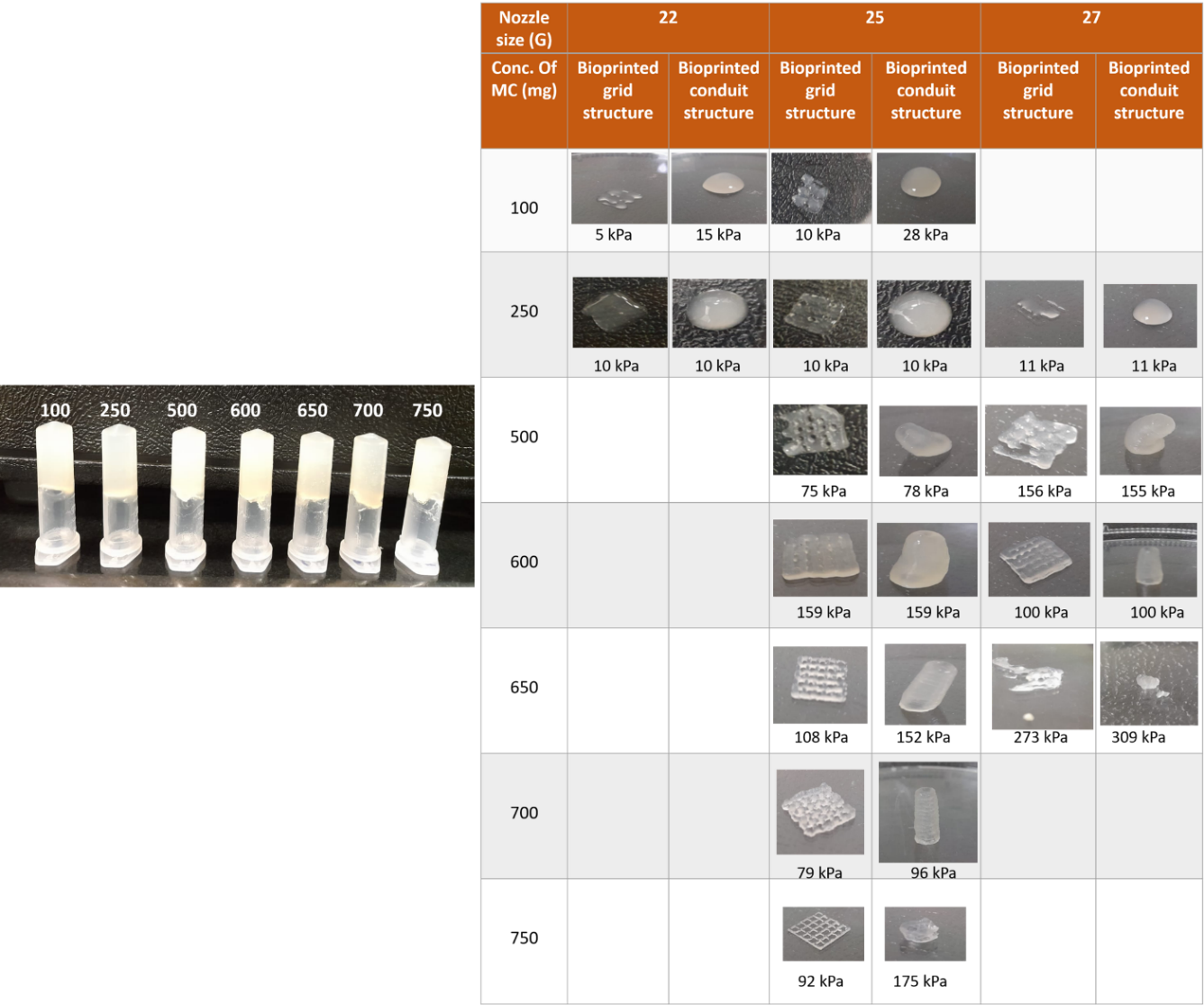


**Figure S2**: Optimization of bioprinting of 500 mg alginate, 36 mg calcium chloride in 10 ml PBS and various concentrations of MC. Non-flow behavior of various concentrations of MC, numbers on eppendorf tubes are in mg (left side image). Optimization of bioprinting of alginate with 36 mg of calcium chloride and various concentrations of MC. At 700 mg of MC, 25G nozzle size, a proper grid structure (79 kPa) and conduit structure (96 kPa) was printed.
